# Precision greenness exposure is associated with lung cancer risk in medicare beneficiaries

**DOI:** 10.1007/s10552-026-02227-z

**Published:** 2026-08-08

**Authors:** Padideh Lovan, Ma Ruixuan, Sara Fleszar-Pavlovic, Julius R. Dewald, Joanna Lombard, Frank J. Penedo, Yalda Zarnegarnia, William Aitken, Yannine Estrada, Scott C. Brown, José Szapocznik

**Affiliations:** 1 Department of Dietetics and Nutrition, Florida International University, 11200 SW 8Th St, AHC5-#328, Miami, FL 33199 USA; 2 Department of Public Health Sciences, Division of Biostatistics, Biostatistics Collaboration and Consulting Core, Miller School of MedicineUniversity of Miami, Miami, FL USA; 3 Sylvester Comprehensive Cancer Center, Miller School of Medicine, University of Miami, Miami, FL USA; 4 Department of Public Health Sciences, Miller School of Medicine, University of Miami, Miami, FL USA; 5 School of Architecture, University of Miami, Coral Gables, FL USA; 6 Departments of Psychology, University of Miami, Coral Gables, FL USA; 7 Kirk Kerkorian School of Medicine, University of Nevada, Las Vegas, NV USA; 8 School of Nursing and Health Studies, University of Miami, Coral Gables, FL USA; 9 Department of Medicine, University of Miami, Sylvester Comprehensive Cancer Center, Coral Gables, FL USA

**Keywords:** Lung cancer, Neighborhood, Greenness, Cancer prevention, Environment, Older adults

## Abstract

**Purpose:**

This study examines whether lung cancer risk is influenced by block-level greenness, controlling for age, sex, race/ethnicity, area deprivation index (ADI), and walkability in older adults, and whether age, sex, race/ethnicity, ADI, or walkability plays a moderating role in this relationship.

**Methods:**

Data from US Medicare beneficiaries aged 65 years and older were included for the years 2011 and 2016. Beneficiaries included in this study received a lung cancer diagnosis for the first time between 2012 and 2016. All resided in Miami-Dade County, Florida (*M*_age_ = 74.31 ± 6.95, 58.69% females, 20.24% White, 68.12% Hispanic, 11.63% Black). Greenness was measured using normalized difference vegetation index. Greenness in 2011 was used to predict lung cancer risk in 2012–2016. Generalized estimating equation (GEE) analyses were performed.

**Results:**

Results indicated that 754 out of 234,360 individuals (0.32%) were diagnosed with lung cancer in 2012–2016. Higher neighborhood greenness in 2011 was associated with lower lung cancer risk from 2012 to 2016 after adjustment for age, sex, race/ethnicity, ADI, and walkability, with each 0.1-unit increase in NDVI corresponding to an approximately 9% lower lung cancer risk (OR = 0.91, 95% CI: 0.85–0.98, *p* = .008). In the best-fit model, walkability moderated the associations between greenness and lung cancer risk, with the beneficiaries living in non-car dependent neighborhoods experiencing a lower risk. In stratified analyses, inverse associations were observed among beneficiaries younger than 75 years, men, Hispanic beneficiaries, and non-car dependent neighborhoods.

**Conclusion:**

Our findings suggest that higher greenness at the Census block level may be associated with reduced lung cancer risk in older adults. This underscores the critical role of environmental factors, particularly access to green spaces, in cancer prevention strategies. Future research should further explore the interplay among biological, behavioral, or social factors that may shape the association between greenness and lung cancer risk.

## Introduction

Lung cancer remains the leading cause of cancer-related mortality in the U.S. and globally, accounting for more deaths than breast, colon, and prostate cancers combined [1]. While smoking tobacco is the most significant and well-established risk factor, accounting for approximately 80–90% of lung cancer cases, [2] increasing attention is being paid to non-smoking-related and environmental risk factors (e.g., air pollution, occupational carcinogens) as well as possible protective factors such as greenness, which may also contribute to lung cancer risk especially in older adults [3–5].

Environmental exposures, such as air pollution, radon, secondhand smoke, and occupational carcinogens (e.g., asbestos, arsenic, diesel exhaust, and polycyclic aromatic hydrocarbons), are well-documented contributors to lung cancer risk and incidence [6–11]. Fine particulate matter, in particular, has been linked to increased lung cancer risk through mechanisms involving chronic inflammation, DNA damage, and oxidative stress [12–14]. Long-term exposure to fine particulate matter has been associated with elevated lung cancer mortality in both smokers and non-smokers, especially in densely populated urban areas where environmental pollution tends to concentrate [7, 13, 15–17]. Radon exposure, a naturally occurring radioactive gas found in soil, rock, water, and buildings, is the second leading cause of lung cancer in the U.S. and the leading cause among non-smokers [8, 18].

In this context, the role of protective environmental factors, such as exposure to green space, has attracted growing interest and the normalized difference vegetation index (NDVI), a satellite-derived measure that compares near-infrared and red-light reflectance to assess vegetation density, is commonly used to assess greenness levels [19]. This objective, scalable metric enables assessment of environmental exposures to greenness across large populations and geographical areas. Numerous studies have linked higher NDVI and neighborhood greenness to reduced all-cause mortality, lower rates of cardiovascular disease, and improved mental health outcomes, including reductions in depression, anxiety, and stress [20–25]. These benefits may be mediated by improved air quality, lower exposure to urban heat, greater opportunities for physical activity and social interaction, and psychological restoration [26]. However, data related to the association between greenness exposure and lung cancer risk remain limited.

Previous research suggests that greenness, such as tree, shrubs, or other ground cover, may mitigate harmful effects of air pollution by filtering airborne pollutants, potentially reducing the risk of pollution-related diseases [27]. For example, Liu and colleagues’ [28] systematic review and meta-analysis found that each 0.1-unit increase in NDVI was associated with a 2–3% reduction in cardiovascular disease mortality, emphasizing the protective cardiovascular benefits of exposure to green environments. In a study examining the 35 most populated metropolitan areas in the U.S., it was estimated that each 0.1 unit increase in NDVI could prevent 15 to 20 deaths per 10,000 individuals aged 65 and older, highlighting the potential impact of urban greening on public health at the population level [29]. Similarly, a recent study reported that each 0.1-unit increase in NDVI within 1,000 m of an individual’s residence was associated with a 16% lower risk of cardiovascular risk [30]. Existing research on the association between NDVI and lung cancer suggests that greater greenness exposure vegetation may mitigate the risk of developing lung cancer [31–33]. Additionally, while greener neighborhoods are not always more walkable [34], both greenness and walkability, independently and in combination, have been found to be associated with increased physical activity [35], which in turn has the potential to reduce the risk of lung cancer [36]. These findings underscore the growing recognition of greenness as a key environmental determinant of health and support the integration of green space into urban design as a health promotion strategy.

Few studies, however, have directly examined the relationship between greenness and lung cancer risk, particularly in older adult populations who may be more vulnerable to environmental risk factors due to cumulative exposure over time and physiological vulnerabilities (e.g., reduced immune function, chronic health conditions, and weakened respiratory system) [37, 38]. Additionally, little is known about the moderating roles of sociodemographic characteristics, including age, sex, race/ethnicity, and neighborhood deprivation, despite existing disparities in both environmental exposure and lung cancer outcomes [29, 39–43].

To address these gaps, the present study investigates whether block-level NDVI scores are associated with lung cancer risk among older adults residing in Miami-Dade County, Florida. Using NDVI data from 2011 and Medicare data from 2012 to 2016, we assessed the effect of greenness on lung cancer risk while adjusting for the beneficiaries’ demographic characteristics (i.e., age, sex, and race/ethnicity) as well as area deprivation index (ADI) and walkability. Furthermore, we examined whether the relationship between greenness and lung cancer is moderated by age, sex, race/ethnicity, ADI, and walkability. Understanding the relationship between precision greenness and lung cancer, particularly through the lens of age, sex, race/ethnicity, ADI, and walkability is critically important because it reveals possible interactions between greenness exposure and social determinants that could shape health outcomes. If greenness is shown to be protective, it not only underscores the health value of urban and suburban vegetation but also highlights areas for policy interventions, since access to green spaces is often unevenly distributed across sociodemographic and neighborhood characteristics. Moreover, this study extends our previous work by uniquely investigating the longitudinal effects of precision greenness measured at the micro-environmental level, defined as the Census block on lung cancer risk. This measure represents a granular neighborhood unit, to capture localized environmental exposures and better assess the direct impact of immediate residential greenness on individuals [44]. By identifying who benefits most from precision greenness, this research can inform focused public health interventions and urban planning policies to reduce lung cancer risk and promote healthier, more resilient communities.

## Methods

### Design

A prospective, longitudinal design compared Medicare beneficiaries in Miami-Dade County living in Census blocks with varying degrees of greenness in 2011 on their lung cancer risk between the years 2012 and 2016.

### Study population

**Fig. 1:**
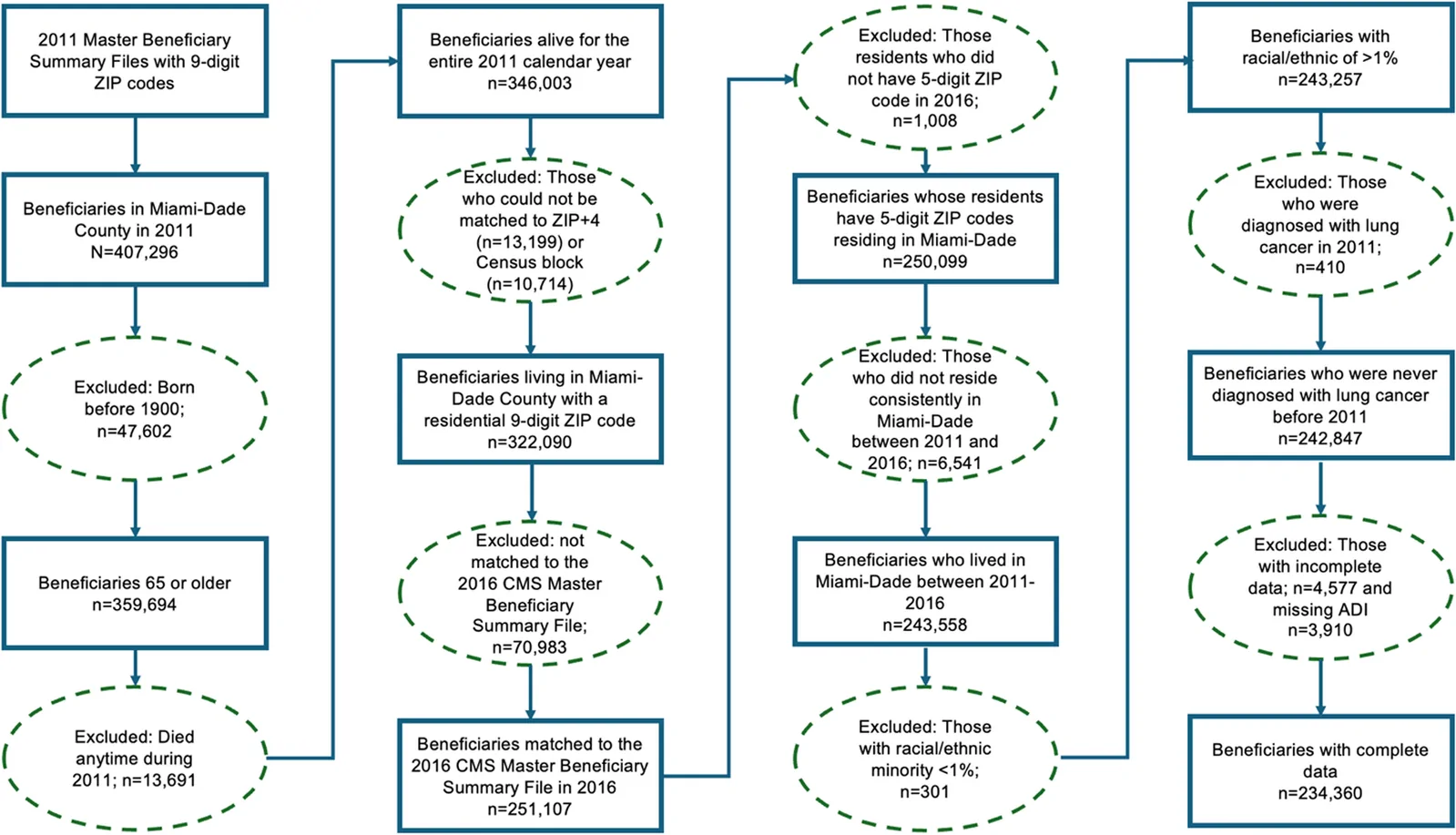
Inclusion and exclusion criteria and the flow of the study sample

The current study uses a sampling procedure similar to that used in our prior studies of chronic disease among Medicare beneficiaries [23, 24]. We analyzed Medicare claims data in 2011 to exclude individuals with lung cancer diagnosis and in 2012 through 2016 to identify lung cancer risk. We only included participants who were in the dataset in 2011 and 2016. First, a cohort was identified that comprised all Medicare Beneficiaries 65 years or older, alive throughout 2011. To identify those who lived in a Census block, ZIP + 4 data from Centers for Medicare and Medicaid Services (CMS) data were linked to a Census block for each beneficiary in 2011 using GeoLytics ZIP + 4 software (GeoLytics, Inc), which provides the area centroid of the ZIP + 4 with latitude and longitude coordinates, and assigns the corresponding Census block and block group identification numbers. Specifically, starting with the 2011 CMS Master Beneficiary Summary File for Miami-Dade County, of 407,296 unique Medicare beneficiaries, the following exclusions were made. We excluded beneficiaries who were younger than 65 or born before 1900 (*n* = 47,602), those who died at any time during 2011 (*n* = 13,691), those whose residence could not be matched to either a ZIP + 4 (*n* = 13,199) or Census block (*n* = 10,714), those who could not be matched to the 2016 CMS Master Beneficiary Summary File (*n* = 70,983; possibly because they had moved out of the study area or had died), and those whose residence did not have 5-digit ZIP code information in 2016 (in which case we did not know if beneficiaries still resided within Miami-Dade County throughout the study period; *n* = 1,008). Then, based on the Medicare Beneficiary Enrollment DataBase, we excluded beneficiaries who did not consistently reside in a Miami-Dade Census block during the entire 2011 to 2016 period (*n* = 6,541), yielding a beneficiary cohort of 243,558. Finally, we excluded 301 beneficiaries belonging to racial/ethnic groups that comprised less than 1% of the Miami-Dade County population, 410 beneficiaries with prevalent lung cancer in 2011, 4,577 beneficiaries with incomplete data, and 3,910 beneficiaries missing ADI information, resulting in a final analytic sample of 234,360 beneficiaries. This study was approved by the University of Miami’s Institutional Review Board and CMS Privacy Board. Figure 1 illustrates the process of applying inclusion/exclusion criteria to determine the final study sample.

### Variables/measures

*Centers for medicare and medicaid services data* Lung cancer determination was made using the lung cancer CMS Chronic Conditions Data Warehouse algorithm (from the Chronic Conditions segment). Data related to 2011 CMS were obtained in 2013, and 2016 CMS data were obtained in 2020 (CMS chronic conditions algorithms are described at https://www2.ccwdata.org/web/guest/condition-categories). Specifically, the lung cancer CMS chronic conditions algorithm defines the presence/absence of lung cancer using particular international classification of diseases (ICD)-9 codes presented in Table 1 (available at https://www2.ccwdata.org/web/guest/condition-categories).

**Table 1:** Lung cancer international classification of disease (ICD)-9 codes

ICD-9 Code	Diagnosis
162.2	Malignant neoplasm of main bronchus
162.3	Malignant neoplasm of upper lobe, bronchus or lung
162.4	Malignant neoplasm of middle lobe, bronchus or lung
162.5	Malignant neoplasm of lower lobe, bronchus or lung
162.8	Malignant neoplasm of other parts of bronchus or lung
162.9	Malignant neoplasm of bronchus and lung, unspecified
231.2	Carcinoma in situ of bronchus and lung
V10.11	Personal history of malignant neoplasm of bronchus and lung

Inclusion criteria: CMS Chronic Condition Warehouse (CCW) Algorithm requires at least 1 inpatient/SNF claim or 2 HOP/Carrier claims with DX code within 1 year

The CMS Chronic Conditions algorithms use claims-based diagnostic codes submitted by clinicians (e.g., physicians, nurse practitioners) and verified by professional coders to determine the presence or absence of each condition. While individual ICD codes can be influenced by documentation and billing practices, these algorithms apply standardized longitudinal criteria (e.g., requiring multiple claims and care settings) to enhance specificity and reproducibility and are widely used in epidemiologic research, with reasonable validity relative to clinical diagnoses. For lung cancer, the algorithm requires at least one inpatient, skilled nursing facility, or home health agency claim, or two hospital outpatient or carrier claims, with a lung cancer diagnosis code within the prior two years. Incident lung cancer identified by this algorithm from 2012 through 2016 was used as the outcome (lung cancer risk) in the present study. We also created a comorbidity score used to control for other comorbidities [45]. Comorbidity information was derived from the Chronic Conditions segment of the CMS Master Beneficiary Summary File, which covers the past 1–3 years, depending on the specific condition. We created a comorbidity count by summing the number of chronic conditions other than lung cancer, yielding a possible range of 0–26 conditions, consistent with our prior work [46, 47]. The Chronic Conditions segment includes indicators for 27 chronic conditions in total, one of which is lung cancer, with nearly all of the remaining 26 representing physical health conditions (e.g., diabetes and cardiovascular disease conditions).

*Location* This study investigates whether the immediate geographic area surrounding an individual's residence (precision greenness), specifically the Census block, is associated with a lower risk of lung cancer. Accordingly, we define the term "micro-environment" as the Census block in which the participant resides, rather than the time–space activity as in other studies [48]. According to the US Census Bureau, Census blocks constitute the smallest geographic area assessed by the U.S. Census and are identified through the ZIP + 4 code [49]. In urban areas, the Census block is typically comprised of a single city block bounded on four sides by streets and avenues. The CMS’ Master Beneficiary Summary File provided person-level data for location (ZIP + 4 for 2011; and the five-digit ZIP code for 2016; ZIP + 4 was not available in the CMS 2016 Master Beneficiary Summary File), new lung cancer condition, age, sex, and race/ethnicity. CMS’ Enrollment Database obtained in 2020 provided the history of known ZIP + 4 s, based on the “Beneficiary Residence Change Date" and “Beneficiary Mailing Contact Zip." The Enrollment Database was used to determine who moved between 2011 and 2016. For non-movers, the ZIP + 4 of 2011 was applied to 2016. For movers, the Enrollment Database provided the ZIP + 4 for 2016.

*Normalized difference vegetation index* NDVI has been established as a well-validated measure of neighborhood greenness in epidemiological research [50, 51], and is a commonly used indicator of vegetative greenness [51]. NDVI has been widely used in previous studies investigating the association between greenness and health [52–55]. NDVI is derived by taking the difference between near-infrared and red reflectance values and dividing it by their sum [56]. This calculation yields a value ranging from − 1 to + 1, where values closer to 0 indicates no vegetation, negative values indicate water, and values closer to + 1 represent dense, healthy vegetation [57]. Areas rich in healthy vegetation absorb a higher amount of red light for photosynthetic activity and reflect a larger portion of near-infrared light resulting in higher NDVI values, whereas areas with low vegetation cover reflect more red light and less NIR, resulting in lower NDVI values [52].

**Fig. 2:**
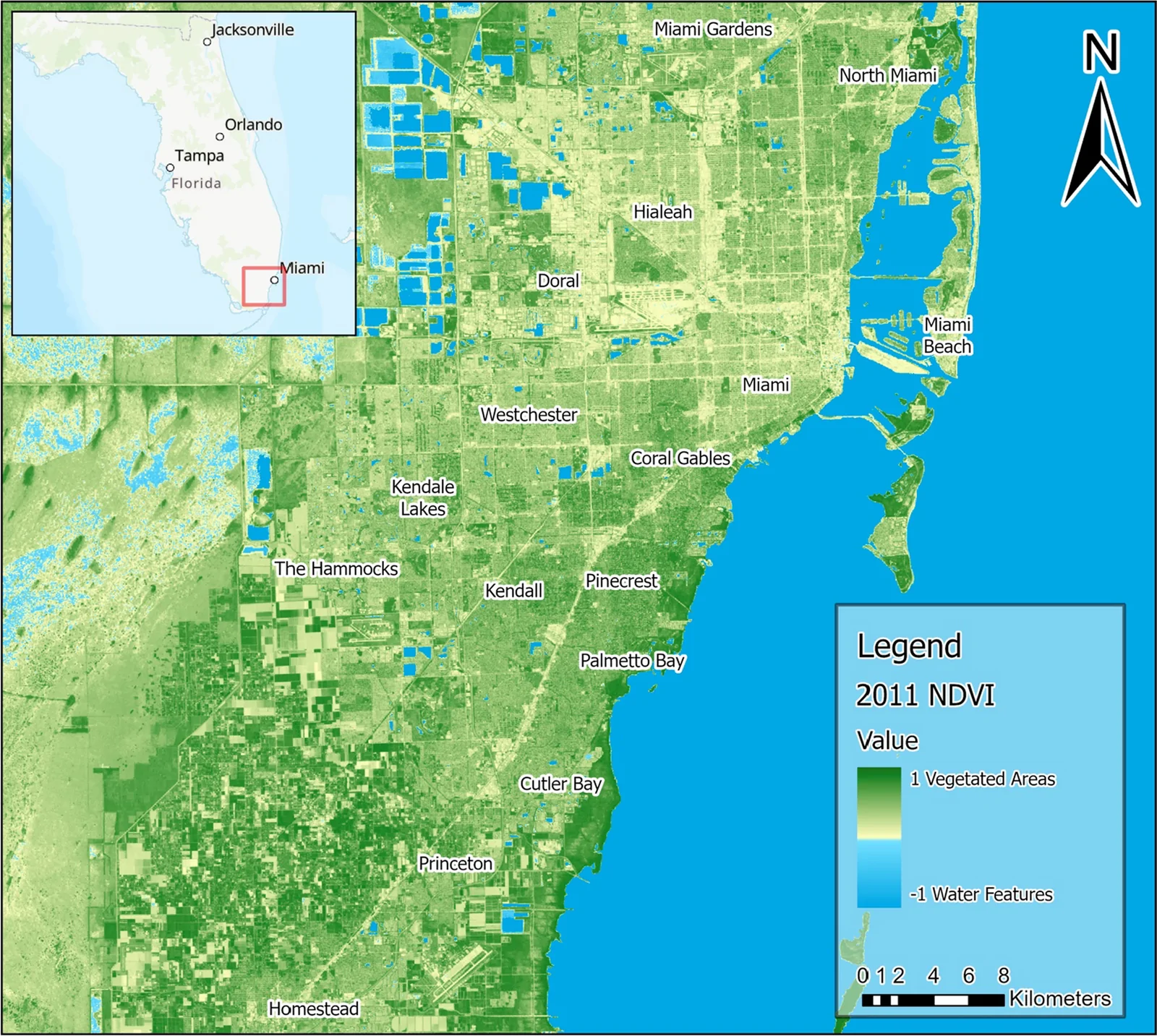
Neighborhood greenness: 2011 NDVI scores at the block level for Miami-Dade County

One challenge in assessing greenness in tropical climates is persistent cloud cover. At the same time, Miami-Dade County exhibits relatively stable year-round greenness. Therefore, we selected a single cloud‑free Landsat 5 scene from 11 November 2011 (30 × 30‑meter resolution), when Miami-Dade County was more than 95% cloud free, to represent typical 2011 neighborhood greenness across the study area and align with baseline residential addresses. This image was obtained from the United States Geological Survey’s earth resources observation and science (EROS) science processing architecture (ESPA) On Demand Interface (Available at https://espa.cr.usgs.gov). We used Level 2 data, which provides atmospherically corrected surface reflectance for the red and near-infrared wavelengths used to calculate the NDVI spectral index. Bodies of water were removed manually. Cloud and cloud-shadow pixels were removed from the images using the pixel QA band [58, 59]. Average NDVI values were calculated for all 23,856 Census blocks in Miami-Dade County where Medicare beneficiaries lived in 2011. Consistent with previous research on NDVI and health outcomes [60], we utilized a 0.1-unit change in 2011 NDVI, a threshold that has been found to be associated with a variety of chronic disease outcomes among Miami-Dade County Medicare beneficiaries [23]. Figure 2 presents the neighborhood greenness calculated by NDVI in Miami-Dade County.

*Area deprivation index (ADI)* We additionally adjusted for neighborhood socioeconomic context using ADI national scores, which captures broader area-level socioeconomic conditions that could confound the association between greenness and lung cancer risk [61, 62]. This validated measure classifies neighborhoods’ socioeconomic distress using data from the US Census Bureau including 17 specific indicators (e.g., housing quality, education, and poverty) [63]. ADI provides an average score of area deprivation using 5-year American Community Survey (ACS) estimates at the Census block group level. Therefore, we used ADI scores related to the year 2010, which represents an average ADI between 2006 and 2010. This measure is freely available to the public at both the state-level and the national-level at https://www.neighborhoodatlas.medicine.wisc.edu/. Additionally, ADI is a reliable measure of socioeconomic conditions for population-based studies [64–66].

*Demographic characteristics* Information on beneficiaries’ age, sex, and race/ethnicity was obtained from CMS data.

*Neighborhood walkability* Walk Score® was used to assess neighborhood walkability. This metric assigns points based on the proximity to various destination types (e.g., retail, recreational) using multiple data sources, including Google and OpenStreetMap [67, 68]. Points are summed and normalized to produce a score of 0–100. The measures demonstrate acceptable levels of reliability and validity [67–69]. Walk Score® data were obtained in 2014 from https://www.walkscore.com, based on calculations conducted in 2013 for the centroid of each Census block in Miami-Dade County. Because Walk Score® did not archive historical data for Miami-Dade County and given that walkability tends to change slowly over time, we linked participants’ residential locations from 2011 to the corresponding 2013 Walk Score® for the centroid of every Census block in Miami-Dade County.

*Covariates* Covariates were individuals’ age, sex, race/ethnicity, ADI, and walkability.

*Moderators* We stratified potential moderators of the relationship between greenness and lung cancer risk as follows: age into "young-old" (65–74 years) and "old-old" (75 years and older); sex into male and female; race/ethnicity into non-Hispanic white, non-Hispanic Black, and Hispanics; ADI using a median split (Median: 36); and walkability (0–49 indicating car dependent; and 50–100 indicating non-car dependent).

### Statistical analysis

All analyses were conducted using SAS software, version 9.4. Univariate descriptive analyses, found in Table 2, were conducted to describe the pattern of individual sociodemographic (age, sex, race/ethnicity), ADI, NDVI, Walk Score, number of comorbidities, and lung cancer risk during the study period (2012–2016) for the overall study sample and the beneficiaries diagnosed with lung cancer.

**Table 2:** Beneficiaries’ demographic and neighborhood characteristics in 2011

Variables	Overall sample	Diagnosed with lung cancer	
	N (%)	N (%)	
N (Beneficiaries)	234,360 (100%)	754 (0.32%)	
Demographic characteristics, %			
Age ≥ 75 years old (%Yes)	43.17%	44.83%	
Gender (%Female)	58.69%	47.21%	
Hispanic (%)	68.12%	59.28%	
Non-hispanic White (%)	20.24%	35.28%	
Black (%)	11.63%	5.44%	
Neighborhood characteristics, Mean (SD)			
NDVI	0.35 (0.11)	0.34 (0.13)	
Area Deprivation Index	36.90 (18.83)	33.88 (20.40)	
Walk Score (2013)	55.11 (17.08)	56.33 (18.85)	
Medical conditions, Mean (SD)			
Comorbidities	1.89 (3.06)		4.48 (3.51)

To test the hypothesis that higher greenness assessed as a continuous variable in 2011 is associated with reduced odds of lung cancer risk from 2012 to 2016, we fit a multivariable generalized estimating equations (GEE) model with exchangeable correlation structure. This approach accounts for within-Census block group correlation in estimates of variability used in tests of significance and estimates population-averaged effects while adjusting for potential covariates. It is important to note that there are 1,594 block groups in Miami-Dade County [70]. The GEE models were fitted using PROC GENMOD to evaluate the association between 2011 NDVI scores and the binary outcome of incident lung cancer diagnosis between 2012 and 2016, adjusting for age, sex, race/ethnicity, ADI, and walkability.

Planned post hoc analyses examined whether age, sex, race/ethnicity, ADI, and walkability scores moderated the relationship between greenness and lung cancer risk. For each potential moderator, cross-level interactions between greenness and the moderator of interest were included in the model, adjusting for all covariates. To maximize model fit, we assessed a series of candidate GEE models with different combinations of interaction terms between greenness and the moderator of interest. The Quasi-Likelihood Information Criterion with Correction for working Correlation (QICu) was the model selection criterion for the GEE approach. The model with the lowest QICu value was selected from the candidate GEE models as the best-fit model.

## Results

This study included 234,360 beneficiaries aged 65 years and older who resided in Miami-Dade County between 2011 and 2016. Mean age was 74.31 ± 6.95 in the overall sample and 74.48 ± 6.43 in beneficiaries diagnosed with lung cancer (0.32% of the overall sample). Detailed descriptive statistics are provided in Table 2.

### Main effect of greenness

The results of the multivariable analyses examined the association between 2011 NDVI values for greenness and lung cancer risk from 2012 to 2016. For each 0.1-unit increase in NDVI, the odds of lung cancer diagnosis were significantly reduced by 9% (OR = 0.909; 95% CI 0.846, 0.976; *p* = 0.008), adjusting for individual age, sex, race/ethnicity, ADI, and walkability.

### Moderators of the impact of greenness on lung cancer

Additional post hoc analyses consisted of a best-fit multivariable model, using the lowest QICu as the model selection criterion. Separate interaction terms were also conducted for the greenness with each moderator including, age, sex, race/ethnicity, ADI, and walkability. We assessed the potential moderating effects both by including all moderators simultaneously in the model and by testing each one individually, to ensure the robustness and consistency of the estimated effect modifications.

### Age

In the best-fit model, the interaction term with age was not statistically significant (β = 0.041, 95% CI − 0.089, 0.172; *p* = 0.537), suggesting that the relationship between greenness and lung cancer risk did not vary meaningfully by age group. However, the interaction-separated model indicated that among beneficiaries younger than 75 years, each 0.1-unit increase in NDVI was associated with an approximately 10% lower lung cancer risk, whereas no significant association was observed among those aged 75 years or older, suggesting that the potential benefits of greenness in reducing lung cancer risk may be more pronounced in younger Medicare beneficiaries.

### Sex

In the best-fit model, the NDVI-by-sex interaction term was not statistically significant (β = 0.115, 95% CI − 0.013, 0.244; *p* = 0.079); however, the interaction-separated model showed that each 0.1-unit increase in NDVI was associated with a 14% lower lung cancer risk among men, with no significant association among women.

### Race/ethnicity (non-hispanic white, non-hispanic black, and hispanic)

In the best model fit, the NDVI-by-race/ethnicity interaction, where non-Hispanic White beneficiaries served as the reference group, the interaction between greenness and Race/Ethnicity was not statistically significant (β = -0.086; 95% CI − 0.493, 0.321; *p* = 0.677). The interaction-separated model showed statistically significant associations for Hispanic beneficiaries (β =  − 0.131; 95% CI − 0.261, − 0.001; *p* = 0.047) indicating that each 0.1-unit increase in NDVI was associated with a 15% lower lung cancer risk among Hispanic beneficiaries. In contrast, no such significant relationship was found for non-Hispanic White or non-Hispanic Black suggesting that the association between greenness and lung cancer risk varies by race/ethnicity.

### Area deprivation index

In the best-fit model, there was no statistically significant evidence of an interaction between NDVI and neighborhood deprivation (dichotomized at the sample median ADI), indicating that neighborhood deprivation did not modify the association between NDVI and lung cancer risk. The NDVI-by-ADI interaction term was not statistically significant (β =  − 0.018, 95% CI − 0.081, 0.045; *p* = 0.574). However, in the interaction-separated model associations were shown to be statistically significant indicating significant associations among both beneficiaries residing in lower-deprivation neighborhoods and among those in higher-deprivation neighborhoods. These findings suggest that the NDVI–lung cancer association may differ across levels of neighborhood socioeconomic deprivation, as characterized by the area deprivation index (ADI).

### Walkability

In the best-fit model, the NDVI-by-walkability interaction was statistically significant (β =  − 0.142, 95% CI − 0.279, − 0.004; *p* = 0.043), and this model improved fit relative to the age-, sex-, and race/ethnicity-interaction models based on the lowest QIC/QICu values. In the interaction-separated model, each 0.1-unit increase in NDVI was associated with a 14% lower lung cancer risk among beneficiaries living in non-car-dependent neighborhoods, whereas no significant association was observed in car-dependent neighborhoods.

### Sensitivity analysis

To assess the robustness of our findings, we conducted a sensitivity analysis restricting the sample to beneficiaries who remained at the same residence from 2011 through 2016. Participants whose 2016 address matched their 2011 address were classified as non‑movers, and those whose 2016 address differed from their 2011 address were classified as movers. The primary analysis reported above included all beneficiaries from both years, regardless of their relocation status. However, residential mobility may introduce potential misclassification of long-term greenness exposure, as individuals who moved could have experienced changes in their environmental surroundings that might influence the observed associations. By focusing on non-movers, we aimed to determine whether the relationship between greenness and lung cancer risk remained consistent when considering only individuals with stable environmental exposure.

Approximately 20% of beneficiaries (46,871 of 234,360) moved during follow-up, whereas 80% (187,489 of 234,360) remained at their baseline address. The results of the sensitivity analysis for non-movers only were largely consistent with those of the primary analysis, reinforcing the robustness of our findings. The inverse association between NDVI and lung cancer risk remained statistically significant in the fully adjusted model controlling for age, sex, race/ethnicity, ADI, and walkability (OR = 0.907, 95% CI 0.837, 0.984; *p* = 0.019), corresponding to a 9% lower risk per 0.1-unit increase in NDVI, suggesting that our results are consistent with previous findings.

In the best-fit model for these moderator analyses for the non-movers only, the interactions of NDVI-by-age (OR = 0.035, 95% CI − 0.113, 0.184; *p* = 0.640), NDVI-by-sex (OR = 0.132, 95% CI − 0.014, 0.279; *p* = 0.077), NDVI-by-race/ethnicity (OR =  − 0.105, 95% CI − 0.257, 0.046; *p* = 0.172 for Hispanic; OR =  − 0.250, 95% CI − 0.652, 0.181; *p* = 0.255 for Black), NDVI-by-ADI (OR =  − 0.030, 95% CI − 0.103, 0.043; *p* = 0.421), and NDVI-by-walkability (OR =  − 0.129, 95% CI − 0.285, 0.026; *p* = 0.103) were not statistically significant. However, stratified estimates suggested lower lung cancer risk associated with higher NDVI among beneficiaries younger than 75 years, men, Hispanic beneficiaries, both lower ADI and higher ADI, and those living in non-car-dependent neighborhoods. These stratified results are best interpreted as descriptive and supportive of exploratory effect modification hypotheses rather than definitive evidence of moderation. Overall, this sensitivity analysis suggests that the association between greenness and lung cancer risk is not strongly driven by residential mobility. The findings remain consistent across models, suggesting that individual demographic characteristics and aspects of the built environment may shape the extent to which greenness influences lung cancer risk.

In our second sensitivity analyses, we added comorbid medical conditions to the models to account for underlying health status and health care use, which may be associated with lung cancer risk and may also be correlated with both neighborhood greenness and covariates such as age and ADI. After adjustment for comorbidities, the inverse association between NDVI and lung cancer risk observed in models without comorbidities was attenuated and no longer statistically significant in the overall sample (OR = 0.950, 95% CI 0.888, 1.017; *p* = 0.146) or in non-movers (OR = 0.955, 95% CI 0.884, 1.032; *p* = 0.249). Because comorbidities likely reflect accumulated effects of long-term behavioral and environmental exposures (for example, smoking, physical inactivity, and cardiometabolic disease), adjusting for them may partially control away the pathways through which greenness could influence lung cancer risk, biasing estimates toward the null [71, 72]. Table 3 presents the summary of all the interaction-separated models for the key subgroups.

**Table 3:** Summary of interaction-separated model estimates for the associations between residential NDVI and lung cancer risk, with multiplicative interaction tests

Moderators; Overall (*n* = 234,360)	Stratum	OR per 0.1-unit higher NDVI	95% CI	*p*-value for interaction-separated NDVI association	*p*-value for NDVI x moderator interaction
Age	Age < 75	0.892	(0.814–0.979)	0.016	0.537
	Age ≥ 75	0.930	(0.841–1.029)	0.162	–
Sex	Male	0.862	(0.786–0.944)	0.001	0.079
	Female	0.967	(0.874–1.070)	0.524	–
Race/Ethnicity	Hispanic	0.853	(0.775–0.940)	0.001	0.047
	Non-Hispanic White	0.973	(0.881–1.075)	0.596	–
	Non-Hispanic Black	0.893	(0.601–1.326)	0.575	0.677
ADI	Lower deprivation	0.915	(0.849–0.986)	0.021	0.574
	Higher deprivation	0.899	(0.829–0.975)	0.010	–
Walkability	Non-car dependent	0.861	(0.787–0.942)	0.001	0.043
	Car dependent	0.993	(0.890–1.107)	0.904	–
Moderators; Non-movers (N = 46,871)	Stratum	OR per 0.1-unit higher NDVI	95% CI	*p*-value for interaction-separated NDVI association	*p*-value for NDVI x moderator interaction
Age	Age < 75	0.893	(0.802–0.993)	0.038	0.640
	Age ≥ 75	0.925	(0.826–1.035)	0.176	–
Sex	Male	0.852	(0.766–0.946)	0.003	0.077
	Female	0.972	(0.868–1.089)	0.629	–
Race/Ethnicity	Hispanic	0.863	(0.769–0.968)	0.012	0.172
	Non-Hispanic White	0.960	(0.860–1.071)	0.465	–
	Non-Hispanic Black	0.747	(0.491–1.135)	0.172	0.255
ADI	Lower deprivation	0.917	(0.842–0.998)	0.044	0.421
	Higher deprivation	0.891	(0.811–0.979)	0.017	–
Walkability	Non-car dependent	0.861	(0.776–0.956)	0.005	0.103
	Car dependent	0.980	(0.868–1.107)	0.755	–

Odds ratios (ORs) and 95% confidence intervals (CIs) are expressed per 0.1-unit increase in the normalized difference vegetation index (NDVI). Estimates labeled as interaction-separated represent the association between NDVI and lung cancer risk estimated within each moderator stratum using adjusted GEE models. *p*-values for interaction-separated associations test the NDVI–lung cancer association within each stratum. *P*-values for NDVI × moderator interaction are derived from multiplicative interaction terms in adjusted GEE models and assess whether the NDVI association differs across moderator strata. For race/ethnicity, non-Hispanic White participants were the reference group

## Discussion

### Main effect of greenness

The overall results of the study indicated that greater block-level greenness was associated with a lower risk of lung cancer in older adults living in Miami-Dade County. Findings from previous studies examining the association between neighborhood greenness and lung cancer risk have been mixed. While some studies report no significant associations between greenness exposure and lung cancer risk [73, 74], others align with our findings, suggesting that exposure to greener environments may be protective [75–77]. Evidence from large cohort studies remains inconsistent as well. For instance, a prospective cohort study of 19,408 individuals in France, with a 27-year follow-up found no statistically significant association between greenness, as measured by NDVI, and the risk of lung cancer [78]. In contrast, a retrospective longitudinal cohort study using data from the Taiwan Cancer Registry (*n* = 407,415) observed a protective effect of greenspace exposure on lung cancer risk over a median follow-up of 10.37 years [33]. Similarly, a cohort study conducted in the Tel Aviv district involving 144,427 participants reported that higher NDVI values were associated with a reduced risk of lung cancer [31].

This variability in findings across studies may be attributed to heterogeneity across studies, including differences in how greenness exposure is defined, the measurement of different types of lung cancer, variations in study design and quality, geographic locations, and the range of adjusted covariates [75, 79]. For instance, Sakhvidi et al. [78], who reported no significant relationship between greenness and lung cancer risk, utilized NDVI to quantify greenness values during the peak vegetation period each year (May to July), and conducted their NDVI calculations using Google Earth Engine. They defined greenness exposure based on NDVI. To explore potential causal pathways linking greenspace to cancer risk, they applied four buffer sizes (100 m, 300 m, 500 m, and 1000 m) around residential addresses and calculated annual average NDVI values within each buffer. This study controlled for a range of covariates, including but not limited to sex, age, body mass index (BMI), smoking status, family status, and fruit and vegetable intake. In comparison, Zhu et al. [75], who reported a significant relationship between greenness and lung cancer risk using the UK Biobank, assessed greenness using both NDVI and the enhanced vegetation index (EVI), averaging values within radial buffers of 300 m, 500 m, 1000 m, and 2000 m around each participant’s residence. The primary exposure metric was the average NDVI/EVI during the summer of the baseline year, with the 500 m buffer serving as the main focus of analysis. Additionally, their study adjusted for various confounders, including age, sex, BMI, education, the Townsend Deprivation Index, alcohol consumption, smoking status, and family history of cancer. Such heterogeneity in exposure definition, buffer sizes, and covariate adjustment may contribute to inconsistencies in the reported associations across studies.

Beyond these methodological differences, it is also important to consider plausible biological pathways linking greenness to lung cancer risk. In line with contemporary models of environmental carcinogenesis, which highlight tumor promotion and clonal selection in an inflamed tissue micro-environment rather than solely direct mutagenesis, neighborhood greenness may influence lung cancer development by altering both environmental and behavioral determinants of inflammation [80]. Green spaces can improve local air quality and reduce heat, attenuate chronic stress and systemic inflammation, and support higher levels of physical activity and other health‑promoting behaviors, which together may shape the pulmonary micro-environment in ways that affect the expansion of pre‑existing mutated clones [81–84]. In the absence of a clearly established direct pathway, these behavioral and environmental mechanisms likely modify the association between greenness and lung cancer risk.

### Age

In age-stratified analyses, higher greenness was associated with approximately 10% lower odds of lung cancer among “young‑old” beneficiaries (65–74 years), whereas no clear association was observed among those aged 75 years and older. Age, however, did not significantly modify the greenness–lung cancer relationship in formal interaction tests, so these apparent differences between age groups should be interpreted as descriptive patterns rather than strong evidence of effect modification. Although there is limited information available on the impact of greenness on lung cancer in different age groups, our findings align with previous research suggesting that the association between neighborhood greenness, as measured by NDVI, is more pronounced among younger than older adults [76]. These findings could be due to longer cumulative exposure window for older adults, where older adults might have accumulated exposures to environmental toxins or smoking earlier in life, potentially diminishing the benefits of recent greenness exposure [85, 86]. Another potential explanation for these findings is differences in time spent outdoors. As individuals age, they are generally less likely to engage in outdoor activities and may prefer to remain indoors due to factors such as physical limitations, concerns about falling, safety issues, or reduced mobility [87].

### Sex

Regarding sex differences, no significant association was observed between greenness and lung cancer risk among females; however, males living in areas with higher greenness had 14% significantly lower odds of developing lung cancer. Some prior studies have reported significant associations between greenness and lung cancer risk and incidence in both males and females [32]. In contrast, others are consistent with our findings, indicating a significant effect in males but not in females [88, 89]. However, recent studies suggest that the gap in sex differences are narrowing [90]. One explanation for our findings could be sex-based differences in immune response, inflammation, and hormonal regulation that may influence how individuals respond to environmental exposures such as greenness. For example, estrogen has complex roles in lung cancer risk and may modulate responses to oxidative stress or air pollutants differently in females [91]. However, the role of sex hormones—particularly estrogen—in the development and progression of lung cancer among females remains controversial and warrants further investigation [92]. Other potential explanations for our findings may include men’s greater likelihood of engaging in outdoor physical activity [93, 94]. In this contexts, neighborhood greenness may offer a stronger protective effect by improving local air quality and mitigating exposure to airborne carcinogens to which men may be more frequently exposed.

### Race/ethnicity

In race/ethnicity-stratified analyses, higher greenness was associated with lower lung cancer risk only among Hispanic beneficiaries (about 15% lower odds), with no clear association in other racial/ethnic groups. Since interaction was not statistically significant, the results should be considered as descriptive. Additionally, because the majority of the population consist of Hispanics (68.12%), it is possible that the sample for the other racial/ethnic groups were not large enough to attain significance. Lung cancer development is influenced by a complex interplay of factors, including environmental exposures (e.g., cigarette smoking), inherited genetic predispositions, and the accumulation of somatic mutations. Although data related to the effect of greenness on lung cancer in different racial and ethnic groups are extremely limited, the existing variation of these contributing factors across different racial and ethnic groups may help explaining the observed disparities in lung cancer risk, mortality, and survival outcomes [95]. A clear example of this disparity is reflected in data from the U.S. cancer registry, which shows a higher risk of malignancies among individuals of African ancestry compared to Caucasian and Asian American populations, is likely driven by socioeconomic disadvantages and reduced access to healthcare [96]. Our findings, particularly the observation that significant associations were detected only among Hispanic participants, may be attributed to the composition of our study sample, which was predominantly Hispanic; therefore, this study may have had greater statistical power to detect these effects within this group. A study from the colorado home energy efficiency and respiratory health (CHEER) discovered that neighborhoods with the lowest levels of greenness and highest levels of air pollution have the highest numbers of Hispanic residents [97]. However, it is important to recognize that the Hispanic population in Miami-Dade is distinct in its composition, making direct comparisons to other Hispanic populations challenging. Another study of children and adolescents aged 6–15 who engaged in moderate to high-intensity activities in and around public parks found that those from predominantly Hispanic neighborhoods in the top quartile experienced 63% higher inhaled doses of NO_2_, an air pollutant, compared to their counterparts in the bottom quartile [98]. These findings highlight the importance of further investigating the impact of greenness on lung cancer risk across diverse racial and ethnic groups to inform more targeted and effective interventions.

### Neighborhood factors (ADI and walkability)

In our neighborhood analyses, we first found that greenness was significantly associated with lung cancer risk within both lower‑ and higher‑deprivation strata defined by ADI. In separate analyses, we observed that the strength of the greenness to lung cancer relationship differed across neighborhoods with varying levels of walkability. Several large cohort and ecological studies have shown that higher residential greenness is associated with lower lung cancer incidence or mortality after adjustment for conventional socioeconomic indicators, including income and neighborhood deprivation measures, suggesting that green environments may confer protective effects [99–102]. Research has shown that low-income communities and communities of color are disproportionately exposed and affected by environmental hazards such as air pollution and degradation, both in terms of frequency and severity [98]. Additionally, low-income populations residing in disadvantaged neighborhoods may experience poorer lung health and lung function compared to higher-income populations living in more advantaged areas [74, 97]. In these contexts, green spaces can provide a stronger protective effect by improving air quality (e.g., filtering pollutants) [103], reducing heat islands (which are more common in economically disadvantaged areas) [104], and providing opportunities for physical activity and stress reduction [105], which are critical for lung health. Thus, consistent with our findings, the presence of greenness in these areas may offer substantial health benefits by mitigating environmental risks and enhancing protective health factors, leading to a reduced risk of lung cancer.

Although our findings also suggest that residents of more affluent neighborhoods do appear to benefit from residential greenness, prior studies have been less consistent in detecting such benefits in advantaged areas [101, 106]. This discrepancy may reflect the fact that affluent neighborhoods already benefit from favorable environmental conditions, including lower levels of air pollution [107] and fewer harmful exposures [108, 109], which can support better overall respiratory health. In these contexts, additional greenness may still contribute to health but its incremental benefits are potentially more difficult to detect empirically because residents are already starting from a comparatively advantaged baseline [110]. Our use of ADI to examine potential modification of the NDVI-lung cancer relationship adds nuance to the existing literature by integrating a multidimensional, neighborhood‑level deprivation index with greenness exposure, helping clarify how social and physical environments may jointly influence lung cancer risk.

Further, our results suggest that higher greenness might be inversely associated with lung cancer risk in non-car dependent neighborhoods. The evidence in the literature with regard to walkability is mixed: while several studies report that more walkable neighborhoods are linked to lower cancer risk, incidence, and mortality, presumably via higher physical activity and healthier weight profiles [111–115], other work suggests that highly walkable urban environments can also coincide with higher air pollution and multimorbidity [116], which may offset or obscure potential benefits for respiratory and cancer risk and outcomes. In addition, recent analyses indicate that specific walking patterns (for example, more frequent or faster walking) may be protective for lung cancer [112], whereas generic measures of neighborhood walkability show more variable associations [117]. One possible explanation for the stronger inverse association between NDVI and lung cancer risk observed in non-car dependent neighborhoods is that residents in these areas are more likely to spend time outdoors walking, accessing destinations, and engaging with their surroundings [117–119], thereby experiencing greater exposure to local environmental conditions. In contrast, residents of car‑dependent neighborhoods often rely more on motorized travel and may spend relatively less time outdoors in their immediate surroundings, potentially reducing ambient environmental exposures and attenuating observable associations between NDVI and lung cancer risk [119, 120]. Taken together, the finding that walkability may moderate the relationship between greenness and lung cancer risk may reflect a balance between the activity‑supportive features of more walkable neighborhoods and competing risks such as traffic‑related air pollution and comorbidities, that may be partially mitigated by higher levels of greenness.

### Strengths and limitations

We acknowledge that this study has several limitations. First, the findings may not be generalizable beyond the study population, as the research was conducted in a single geographic region. Second, we did not differentiate between lung cancer subtypes or stages, which may have distinct etiologies and environmental sensitivities. Additionally, because diagnosis timing was available only at a claims-based resolution and all beneficiaries shared a common 2011–2016 follow‑up window, we modeled lung cancer as an incident binary outcome rather than using survival or Fine-Gray competing‑risks methods, and we note this as a methodological limitation. Third, lung cancer is a multifactorial disease influenced by lifestyle and personal factors, including smoking status, genetic predispositions, air quality, and broader social and environmental conditions. However, our analysis was limited by the available data: we lacked information on individual lifestyle, smoking history, and air pollution at the same spatial resolution, block level, we used in this study, therefore could not adjust for these confounders. As a result, our relatively modest greenness–lung cancer associations may be affected by unmeasured and residual confounding and should be interpreted with caution, underscoring the need for future studies with richer covariate and air pollution data. Another limitation is that NDVI captures vegetative density and greenness, but not the type, accessibility, usability, or quality of greenspace, which may be important dimensions for lung cancer risk. In this study greenness was defined from a single 2011 Landsat image linked to baseline addresses rather than modeled as a time‑varying exposure. Future work should incorporate richer greenness metrics and time‑varying exposure. Lastly, beyond the movers/non‑movers sensitivity analysis, we lacked detailed information on prior residential histories or duration at the baseline address, limiting our ability to assess how well the 2011 NDVI measurement reflects long‑term greenness exposure given the long latency of lung cancer.

Despite these limitations, this study has several notable strengths. It is among the first to investigate the relationship between precision greenness, measured at the Census block level, and lung cancer risk in South Florida, an area with distinct environmental and demographic characteristics. We incorporated a comprehensive set of moderating factors to better understand these relationships, offering a more nuanced investigation of for whom greenness may have it greatest impact than has been previously reported. Moreover, the use of Medicare data provided a large, reliable, and well-characterized cohort, enhancing the robustness and credibility of our findings. Furthermore, the use of data available throughout the country, including Medicare, satellite greenness images to estimate greenness, and Census block group-level data, increases the opportunity for replication of this study in other U.S. localities. This work contributes to the growing body of evidence on environmental determinants of cancer and highlights important areas for future research. It is also important to note that in this Medicare cohort, beneficiaries were followed from 2012 to 2016 to assess lung cancer risk, recognizing that this approximately 5‑year window captures only part of the long etiologic latency for lung carcinogenesis. Lung cancer diagnoses were ascertained from claims‑based algorithms, and we modeled lung cancer as an incident binary outcome over 2012–2016, while noting that future work linking to cancer registries could evaluate time‑to‑event outcomes and survival in more detail.

## Conclusion

Our findings suggest that, overall, higher levels of precision block-level greenness are associated with a lower risk of lung cancer among older adults residing in South Florida, and these effects were particularly noted among young-old, males, Hispanics, both high- and low-deprived areas, and beneficiaries living in non-car dependent neighborhoods. This underscores the critical role of environmental factors, particularly access to green spaces, in cancer prevention strategies. Given these findings, future research should further explore the mechanisms through which greenness may influence lung cancer risk, particularly in vulnerable subgroups. Longitudinal studies incorporating more detailed individual-level data, such as smoking history, occupational exposures, duration of residential stability, and physical activity patterns, are needed to better elucidate causal pathways. Additionally, future work should investigate the role of greenspace quality, vegetation types, and usage patterns, to augment the use of NDVI as a proxy for greenness exposure. Expanding research across diverse geographic regions and including racially and ethnically diverse cohorts would also enhance generalizability. Finally, integrating measures of indoor environmental quality, psychosocial stress, and access to healthcare could offer a more comprehensive understanding of how neighborhood environments interact with personal and social factors to influence lung cancer risk.

## Data Availability

No datasets were generated or analysed during the current study.
